# *Tabebuia avellanedae *naphthoquinones: activity against methicillin-resistant staphylococcal strains, cytotoxic activity and *in vivo *dermal irritability analysis

**DOI:** 10.1186/1476-0711-5-5

**Published:** 2006-03-22

**Authors:** Eliezer Menezes Pereira, Thelma de Barros Machado, Ivana Correa Ramos Leal, Desyreé Murta Jesus, Clarissa Rosa de Almeida Damaso, Antonio Ventura Pinto, Marcia Giambiagi-deMarval, Ricardo Machado Kuster, Kátia Regina Netto dos Santos

**Affiliations:** 1 Instituto de Microbiologia Prof. Paulo de Góes – Universidade Federal do Rio de Janeiro, Rio de Janeiro, Brazil; 2 Departamento de Tecnologia Farmacêutica – Universidade Federal Fluminense, Rio de Janeiro, Brazil; 3 Núcleo de Pesquisas de Produtos Naturais – Universidade Federal do Rio de Janeiro, Rio de Janeiro, Brazil; 4 Instituto de Biofísica Carlos Chagas Filho – Universidade Federal do Rio de Janeiro, Rio de Janeiro, Brazil

## Abstract

**Background:**

Methicillin-resistant *Staphylococcus aureus *(MRSA) and coagulase-negative staphylococcus infections are a worldwide concern. Currently, these isolates have also shown resistance to vancomycin, the last therapy used in these cases. It has been observed that quinones and other related compounds exhibit antibacterial activity. This study evaluated the antibacterial activity, toxicity and *in vivo *dermal irritability of lapachol extracted from *Tabebuia avellanedae *and derivatives against methicillin-resistant staphylococcal isolates. In addition, its mechanism of action was also analyzed.

**Methods:**

The compounds β-lapachone, 3-hydroxy β *N *lapachone and α-lapachone were tested to determine the MIC values against methicillin-resistant *S. aureus*, *S. epidermidis *and *S. haemolyticus *strains, being the two last ones hetero-resistant to vancomycin. Experiments of protein synthesis analysis to investigate the naphthoquinones action were assessed. *In vitro *toxicity to eukaryotic BSC-40 African Green Monkey Kidney cell cultures and *in vivo *primary dermal irritability in healthy rabbits were also performed.

**Results:**

The compounds tested showed antibacterial activity (MICs of 8, 4/8 and 64/128 μg/mL to β-lapachone, 3-hydroxy β N lapachone and α-lapachone, respectively), but no bactericidal activity was observed (MBC > 512 μg/mL for all compounds). Although it has been observed toxic effect in eukaryotic cells, the compounds were shown to be atoxic when applied as topic preparations in healthy rabbits. No inhibition of proteins synthesis was observed.

**Conclusion:**

Our results suggest that quinones could be used in topic preparations against wound infections caused by staphylococci, after major investigation of the pharmacological properties of the compounds. Studies about the use of these compounds on tumoral cells could be carried on, due to their effect in eukaryotic cells metabolism.

## Background

The south-american tree *Tabebuia avellanedae *(Bignoneaceae) is known in the popular medicine as *Ipê-Roxo, Pau D'Arco, Lapacho*, among others [1,2]. For many decades, preparations made with this plant were used in South and North America as antineoplasic, antifungal, antiviral, antimicrobial, antiparasitical and anti-inflammatory treatment [1-5]. Pharmacological activities of this species are related to saponins, flavonoids, coumarins, and natural antibiotics [3,6], while the chemical profile presented by most of the studies has shown the quinones as the main active substances [1-4,6].

The increasing prevalence of multi-resistant bacteria made the search of new antimicrobial agents an important strategy for the establishment of alternative therapies in difficult handling infections [3]. Methicillin-resistant staphylococci infectionsmainly caused by *Staphylococcus aureus *(MRSA strains) and by coagulase-negative staphylococci (CNS), as *S*. *epidermidis *(MRSE) and *S. haemolyticus *(MRSH) isolates have increased in the last two decades [7]. They are the pathogens most frequently isolated from nosocomial bacteraemias [8], with an attributable mortality rate ranging from 13% for CNS [9] to 42% for MRSA [11]. In these cases, the therapy is generally limited to the use of vancomycin and teicoplanin. However, some *Staphylococcus *strains resistant to glycopeptides have been reported in Brazil [11] and other countries [12]. Then, the research on new antimicrobial agents is an area of great importance [5,13].

Several naphthoquinones are found in the nature showing activity against aerobic and anaerobic bacterial species. In general, they are active against *S. aureus*, *Enterococcus faecium *and *Bacillus subtilis*, but inactive against Gram-negative bacteria [5]. Its mechanism of action has not been completely elucidated. The naphthoquinone β-lapachone, for example, seems to increase the generation of superoxide anion and hydrogen peroxide in *Trypanosoma cruzi *[14].

In a previous study we described that lapachol derivatives from *Tabebuia avellanedae *showed growth inhibitory activity against MRSA isolates [3]. The aim of the present study was to evaluate the antimicrobial activity of these drugs against multi-resistant staphylococci isolates, including coagulase-negative staphylococcal strains presenting vancomycin-heterogeneous resistance, and to verify *in vitro *toxicity to eukaryotic cell cultures and *in vivo *primary dermal irritability. In addition the mechanism of naphthoquinones action was also investigated.

## Methods

### Bacterial strains

Standard strains of *S. aureus *ATCC 29213 (Methicillin-sensible *Staphylococcus aureus*) and ATCC 33591 (Methicillin-resistant *Staphylococcus aureus*), and the methicillin-resistant clinical isolates *Staphylococcus epidermidis *228 (MRSE) and *Staphylococcus haemolyticus *(225) presenting hetero-resistance to vancomycin and identified in previous study [15] were used. The clinical strains were isolated from bloodstream of patients from a tertiary hospital in Rio de Janeiro city, Brazil. The isolates of *S. epidermidis *were characterized as hetero-resistant to vancomycin through vancomycin agar screening test according to the National Comittee for Clinical Laboratory Standard (NCCLS) [16] and by evaluation of the population analysis profile (data not shown). All organisms were plated on 5% sheep blood agar base (Oxoid) at 35°C for 24 h.

### Eukaryotic cells

BSC-40 cells from African green monkey kidney were propagated in Dulbeccos's modified Eagles's medium (DMEM; Invitrogen) supplemented with 8% calf serum, 2% heat-inactivated bovine serum (BRL/Gibco Laboratories), 50 μg mL^-1 ^gentamicin sulfate, 500 U mL^-1 ^penicillin, 100 μg mL^-1 ^streptomycin, 225 μg/mL sodium bicarbonate and 2,5 μg mL^-1 ^fungizon. Cells were grown as adherent cultures at 37°C in a 5% CO_2 _incubator [17].

### Naphthoquinones

**Figure 1:**
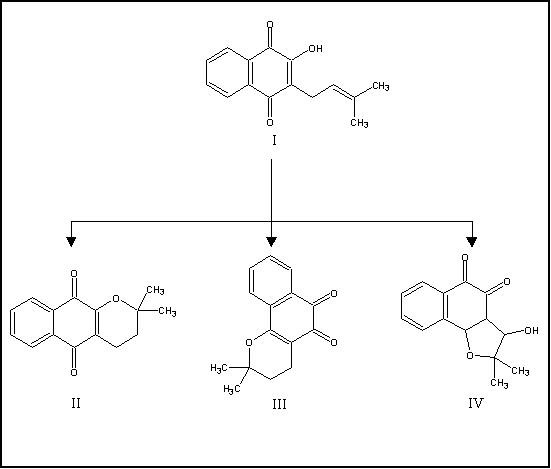
**Naphthoquinones evaluated in this study**. I) Lapachol (*2-hydroxy-3-(3-methylbut-2-enyl)naphthoquinone*); II) α-lapachone (*2,2-dimethyl-2H-benzo [g]chromene-5,10-dione*); III) β-lapachone (*2,2-dimethyl-3,4-dihydro-2H-benzo [h]chromene-5,6-dione*); IV) (±) 3-hydroxy-β-*N*-lapachone ((±)*3-hydroxy-2,2-dimethyl-2,3,3a,9b-tetrahydronaphtho [1,2-d] furan-4,5-dione*)

The naphthoquinones derivatives evaluated in this study (Figure 1) were obtained from the lapachol, which was isolated by extraction from *T. avellanedae *sawdust [18]. The reactions were carried out in the laboratory of organic synthesis of the Natural Products Research Nucleus at UFRJ (NPPN-UFRJ). The α-lapachone, that we named as compound I was synthesized according to Hooker [19] and the other quinones (compounds II, II and IV) were synthesized according to Pinto and coworkers [20].

### Minimal Inhibitory Concentration (MIC) Determination

The MIC was evaluated by the dilution method in Mueller-Hinton broth medium (Oxoid), according to NCCLS [16], for each one of the naphthoquinones, with concentrations ranging from 2 to 512 μg/mL. Bacteria (10^4 ^CFU/mL) were inoculated in the broth with the drug, and incubated at 35°C for 24 h.

### Minimal Bactericidal Concentration (MBC) Determination

MBC is the smaller concentration of the drug necessary for elimination of 99.9% of the microorganisms tested. The MBC was determined after the MIC assays. Tubes where the MIC results showed no bacterial growth, an aliquot of 0.1 mL was seeded in Mueller-Hinton agar without addition of drugs and the bacterial growth was evaluated for the MBC determination. After 24 h, at 35°C, if MIC = MBC or if MBC is one, two or three dilutions above of MIC, the drug is considered bactericide [21].

### Protein synthesis analysis by SDS-PAGE

An overnight culture of *S. aureus *ATCC 33591 was diluted in BHI to 10^7 ^CFU/mL and incubated for 30 min at 37°C. For labeling, cells were concentrated to 10^8 ^CFU/mL in a methionine-free medium (MEM, Gibco) containing 200 μCi/mL of [^35^S] methionine (Amershan) and subject to drug addition, compound IV or menadione (vitamin K3) [22] at final concentrations of 8, 16 and 32 μg/mL at 37°C, or heat treatment at 45°C [23]. In all the tests, the cells were pulse-labeled for 30 minutes and collected by centrifugation at 12,000 g for 5 min. The cells were lysated with the addition of 40 ng/mL of lysostaphin (*S. aureus*) during 2 hours at 37°C. After incubation period, equal volumes of 0.5 M Tris-HCl (pH 7.2) buffer containing 4% SDS, 10% β-mercaptoethanol, 20% glycerol and 0.1 % bromophenol blue were added, and the samples were boiled for 5 min. Cellular extracts were subjected to Polyacrylamide Gel Electrophoresis (SDS-PAGE) analysis. The gel was stained with Comassie-blue, destained, dried and exposed to X-ray films.

### Cell viability by Neutral Red test

The neutral red assay is based on the incorporation of the supravital dye neutral red into living cells. Confluent monolayers of BSC-40 cells (96-well plate) were incubated with a specified concentration of the naphthoquinones for 24 h at 37°C. The control was performed in absence of drugs. Neutral red stock solution (0.1%) was prepared in deionized water and stored at room temperature. Before staining, a fresh 1:100 dilution of the dye was prepared. In accordance to Thompson (1998) [17], 100 μl/well of medium containing neutral red were added to living cells (50 μg/ml final concentration), and the microplates were incubated at 37°C in moist atmosphere with 5% CO_2 _for 3 h. The cells were then washed with 4% formaldehyde and incubated at room temperature for 1 min. After formaldehyde discarding, methanol solution (50%) was added and incubated at room temperature for 20 min. The optical density at 490 nm was measured using a microtiter plate spectrophotometer. The uptake of neutral red is proportional to the number of viable (live) cells [21].

### Primary dermal irritability test

This test was performed according to Draize (1944) [24]. Different concentrations of the naphthoquinones were prepared, according to Table 1. Ten healthy rabbits were selected for each drug solution and the animals separated for chamber adaptation 48 hours before the assay. The animals had not alimentary restrictions and periods of dark and light were intercalated in each 12 hours. The ambient temperature was maintained at 25 ± 2°C. The animals were depilated on the dorsal region 24 hours before the assay. The dorsal region was divided in two parts: the right side, with two limited areas with no blooding chases, and the left one, with two limited areas with intact skin. The concentrations of the alcoholic solutions of the naphthoquinones related to the MIC obtained (Table I) were applied on the pre-established limited areas of the animals. The compound IV was also tested in the concentration of 0.8 mg/mL (MIC 100×). The rabbits were in contact with the solution of the naphthoquinones during four hours, and observations were done during 24, 48, 72 and 96 hours. During this period, the appearance of inflammatory reactions (edema and/or erythematic areas) or any other toxic reactions due to the substances was evaluated. The signals and symptoms observed were classified in agreement with the Federal Hazardous Substances Act of the United States.

**Table 1:** Antimicrobial activity of naphthoquinones against *Staphylococcus *species.

Compounds	*S. aureus *ATCC 29213 (MSSA)		*S. aureus *ATCC 33591 (MRSA)		*S. epidermidis *MRSE 228		*S. haemolyticus *MRSH 225	
	
	MIC (μg/mL)	MBC (μg/mL)	MIC (μg/mL)	MBC (μg/mL)	MIC (μg/mL)	MBC (μg/mL)	MIC (μg/mL)	MBC (μg/mL)
I^a^	256	>512	256	>512	ND	ND	ND	ND
II	64	>512	64	>512	128	>512	128	>512
III	8	>512	8	>512	8	>512	8	>512
IV	8	>512	8	>512	4	>512	8	>512

ND → not determined. Compounds: I) Lapachol; II) α-lapachone; III) β-lapachone; IV) (±) 3-hydroxy-β-*N*-lapachone. MIC = Minimal Inhibitory Concentration MBC = Minimal Bactericidal Concentration

## Results

### MIC and MBC determination

The MIC and MBC determination was performed to compare the antimicrobial effect of the naphthoquinones in MSSA and MRSA strains (*S. aureus*) and evaluate this effect in resistant coagulase-negative staphylococci (*S. epidermidis *and *S. haemolyticus*). The antimicrobial activity of the compounds against *S. aureus *(ATCC 29213 and ATCC 33591), *S. epidermidis *MRSE 228 and *S. haemolyticus *MRSH 225 isolates are shown in the Table 1. Compounds III and IV showed the best results, inhibiting the growth of all bacteria at concentration of 8 μg/mL. For the isolate *S. epidermidis*, the compound IV showed a MIC of 4 μg/mL. A MBC above 512 μg/mL was seen for all the compounds tested, indicating an antibacteriostatic activity.

### Protein synthesis analysis

**Figure 2:**
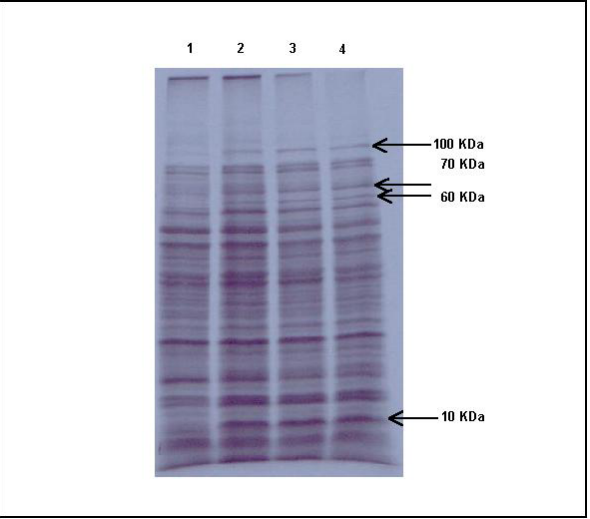
**Analysis of proteins synthesis**. Autoradiogram of a SDS-PAGE protein profile of the *S. aureus *strain ATCC 29213 labeled in the presence of [^35^S] Met (200 μCi/mL) for 30 min at 37°C (lane 1) or at three concentrations of compound IV (±) 3-hydroxy-β-*N*-lapachone (8, 16 and 32 μg/mL – lanes 2, 3 and 4). The arrows on the right indicate the induced proteins and their molecular weight in kDa.

To investigate a possible mechanism of action of naphthoquinones on the bacteria protein synthesis, the compound IV were selected to perform this analyze since it presented the lowest MIC. So, (±) 3-hydroxy-β-*N*-lapachone (compound IV) were added to the staphylococcal cells at different concentrations (8, 16 and 32 μg/mL) in the presence of ^35^S-Met as described in Materials and Methods. After SDS-PAGE, the corresponding autoradiogram showed that this compound did not inhibit the bacterial protein synthesis at the tested concentrations but induced the expression of some proteins of 100, 70, 60 and 10 KDa (Figure 2). The same pattern of induction was observed when cells were submitted to 45°C. We have previously identified these proteins as heat-shock proteins (HSPs) also known as stress proteins [23]. In order to determine if the cellular stress caused by the compound IV could be related to oxidative stress, the menadione (vitamin K3) [22], a well known oxidative stress agent, was tested at 8, 16 e 32 μg/mL, showing the same pattern of induction (data not shown). These results showed that the naphthoquinones analyzed cause a stress reaction in bacterial cell, suggesting that it could be related to oxidative stress.

### Cytotoxicity

**Figure 3:**
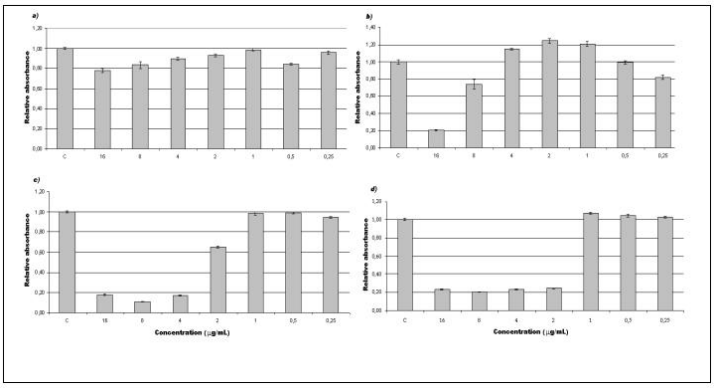
**Citotoxicity assay by the neutral red incorporation method**. Eukaryotic cells (BSC-40) were grown as adherent culture in a 96-well microplate. The compounds were added at concentrations showed in the graphics (16 to 0.25 μg/mL) for 24 h. No drug was added in the first well (control test). The relation between sample absorbance and control absorbance calculated the relative absorbance. Compounds: *a*) I; *b*) II; *c*) III; *d*) IV. The measurements are expressed as average of replicates.

The cytotoxicity was evaluated to determine the toxic concentration of these compounds and compare with the antibacterial concentration observed (MIC), for further analysis of their application in antimicrobial therapy. The compounds presented a considerable cytotoxicity. A concentration of 2 μg/mL of the compound IV was sufficient to kill 80% of the cell culture, while its minimal concentration to inhibit the bacteria was 8 μg/mL. It was observed that the other compounds are less toxic than compound IV. The precursor (compound I) did not present a severe toxicity to BSC-40 cells when compared to the other compounds used at the same concentration (Figure 3). The minimal inhibitory concentrations of the other substances tested with eukaryotic cells are listed in the Table 2. This effect on BSC-40 cells suggests that these compounds could be used in antineoplasic therapy or antibacterial therapy as topic preparations.

**Table 2:** Comparative activity of the compounds against bacterial and eukaryotic cells

Compound	Minimal Inhibitory Concentration^a ^(μg/mL)	Minimal Toxic Concentration^b ^(μg/mL)
I	256	>16
II	64	16
III	8	2
IV	8	2

Compounds: I) Lapachol; II) α-lapachone; III) β-lapachone; IV) (±) 3-hydroxy-β-*N*-lapachone

^a ^MIC – assayed with MRSA strain (ATCC 33591); ^b ^assayed on BSC-40 cells

### Primary dermal irritability test

This assay was performed to determine the toxicity of these compounds (previously considerable a toxic substance after the cytotoxicity determination) in topic preparations. No damage was observed on the limited dermal areas of the animals evaluated at the periods of 24, 48, 72 and 96 hours after the application of naphthoquinones solutions in all concentrations used, including a concentration 100× higher than the MIC found for the compound IV (Table 3). Then, these compounds did not show dermal irritability when used in topic preparations.

**Table 3:** Concentrations of the naphthoquinones used in dermal irritability test

Compound	MIC	100× MIC
I	0.03 % (256 μg/mL)	ND
II	0.01 % (64 μg/mL)	ND
III	0.001% (8 μg/mL)	ND
IV	0.001% (8 μg/mL)	0.1 % (800 μg/mL)

N.D. → not determined. MIC = Minimal Inhibitory Concentration. Compounds: I) Lapachol; II) α-lapachone; III) β-lapachone; IV) (±) 3-hydroxy-β-*N*-lapachone.

## Discussion

The search for new antimicrobial agents is of great concern today, because of the multiple drugs resistance acquired by several pathogens [7]. Currently, in Latin America, the methicillin resistance rates are higher than 40% among *S. aureus *isolates and they are above 70 % among CNS isolates [8]. These strains present the *mecA *gene that encodes a low antibiotic-affinity penicillin-binding protein [7]. Normally, these multi-drug resistant strains are susceptible only to vancomycin [12]. However, the large use of this antimicrobial in hospitals has favored the emergence of vancomycin resistant species, including *S. aureus *[12], *S. epidermidis *and *S. haemolyticus *[11]. Then, the research of new drugs is interesting.

In this study, bacterial growth inhibition by naphthoquinones showed that the synthetic compounds II (α-lapachone), III (β-lapachone) and IV [(±) 3-hydroxy-β-*N*-lapachone] were more effective than their precursor lapachol (compound I), mainly the compounds III and IV. Structural analysis of the compound IV shows that its higher toxic and antimicrobial action could be associated with a hydroxyl group (OH) inserted at furan ring (Fig. 1), as well as related to the naphtho 1,2-quinoidal system that is present in both compounds III and IV, making them more effective than the other compounds tested against the MRSA, MRSE and MRSH isolates. The results show that these naphthoquinones have considerable activity against staphylococci (MICs from 4 to 128 μg/mL), as we have previously reported [3], although the activity presented by the compounds has been bacteriostatic (MBCs > 512 μg/mL). Naphthoquinones activity was observed even against vancomycin hetero-resistant isolates, suggesting that they could be an alternative antimicrobial agent in therapeutic of multi-resistant staphylococcal infections.

Bacterial protein synthesis was not inhibited by naphthoquinones, as it was demonstrated by SDS-PAGE analysis. However, it was observed that some proteins of molecular weight of 100, 70, 60 and 10 KDa, referred as stress proteins, had their levels increased by heat [23] and by menadione treatment [22]. As these proteins are associated with bacterial stress, naphthoquinones could have induced an oxidative stress in all microorganisms, since this class of substance is involved with cell toxicity. It is supposed that these substances promote an oxidative stress in the membrane of the bacterial cell when ATP synthesis occurs in the respiratory chain [25]. Quinones are coenzyme Q analogs (ubiquinone, an electron-transfer substance that carries out the electrons of reduced NAD of the complex I to III), competing with these substances. When the respiratory chain is affected, several reactive-radicals like hydrogen peroxide, hydroxyl radical and superoxide anion have an increase in their concentrations, causing an oxidative stress [25,26]. So, the results here presented show that the naphthoquinones analyzed cause a stress reaction in bacterial cell and suggest that it could be related to oxidative stress.

When the compounds were tested in eukaryotic cells (BSC-40), a cytotoxic effect was observed. The data in Table 2 shows that the toxic concentration of all compounds to the eukaryotic cells corresponded to a quarter of their MIC values. These results show that these substances cannot be used by endogenous administration once they could lead to damage to human cells. However, when the substances were applied as a topic preparation in rabbits, no damage was observed, even when it was used at high concentrations, as seen for the compound IV tested in a concentration 100× higher (800 μg/mL) than the MIC observed for it. These results show that it would be possible to propose a topic use for this compound, either in prophylactic procedures or in the treatment of wound infections after major investigation in relation to a long term application on the derm and blood levels of the compounds.

Some authors describe naphthoquinones with a large antitumoral activity [2,27]. In this study, although the compounds had presented a high toxic concentration in normal epithelial cells, a therapy with these substances would be possible once the tumor cells have a large metabolic rate. Naphthoquinones would be metabolized firstly by neoplasic cells, acting like other toxic chemotherapeutic agents as base analogs, alquilants, and others used in the antitumoral therapy [2,27].

In this study a relationship between structure and toxicity of the different naphthoquinones was observed. Table 2 shows that the substances with lower MIC (4 – 8 μg/mL) presented the highest toxicity (compounds III and IV), while the compound I with a higher MIC (256 μg/mL) did not show any toxic effect at the tested concentrations. It can be considered that structural modifications had a pronounced effect in the activity of the substances. Further studies involving structural modifications will be necessary to decrease their toxicity to eukaryotic cells, maintaining or increasing the antibacterial activity.

## Conclusion

In conclusion, naphthoquinones are an interesting class of natural compounds with antibacterial activity, and could be used mainly as topical drugs against staphylococcal infections, after major investigation of the pharmacological properties of the compounds However, due to the cell toxicity shown for many representatives of them, structural modifications can be an important strategy to produce new molecules, less toxic, to be used on tumor cells, due to the fact these substances present a large effect in eukaryotic cells metabolism.
